# Walk a mile in my old shoes: an intervention study on age simulation suit training for correctional staff

**DOI:** 10.1093/geroni/igag075

**Published:** 2026-06-29

**Authors:** Diete Humblet

**Affiliations:** Faculty of Law and Criminology, Department of Criminology, Vrije Universiteit Brussel, Brussel, Belgium

**Keywords:** Experiential learning, Ageism, Prison

## Abstract

**Background and Objectives:**

Correctional populations are ageing rapidly, yet correctional staff often lack the knowledge and attitudinal competencies required to address ageing-related needs. While simulation technologies, such as simulation suits, have demonstrated improvements in cognitive and affective learning outcomes within educational and clinical healthcare settings, their application in correctional training remains unexplored. This study examined the feasibility and exploratory outcomes of a training program incorporating ageing simulation into correctional staff training.

**Research Design and Methods:**

A mixed-methods pilot study was conducted with 19 correctional officers working with older incarcerated individuals in Flanders (Belgium) and included experiential learning using the GERT ageing simulation suit. Quantitative pre-post survey data were collected to explore changes in knowledge, attitudes toward older adults, willingness to work with this population, and ageism; qualitative focus groups provided in-depth insights into participants’ experiences.

**Results:**

Quantitative findings showed limited and variable changes, consistent with the small sample size and exploratory design. However, qualitative findings indicated that the intervention facilitated reflection on ageing-related physical and psychosocial challenges and increased awareness of the lived experience of ageing in prison. Notably, reluctance to engage with the ageing simulation emerged as an important finding, suggesting that emotional and professional resistance may shape engagement with experiential learning.

**Discussion and Implications:**

Rather than directly changing attitudes, ageing simulation appeared to function primarily as a catalyst for reflection. These findings highlight the importance of contextual and cultural factors in implementing experiential training in correctional settings and provide initial insights to inform the design of future interventions.

Innovation and Translational Significance: This study explores the use of ageing simulation as a promising approach to enhance ageing awareness in correctional training. Findings indicate variable engagement, including resistance to the simulation and role-dependent learning experiences. Rather than producing uniformly more positive attitudes, the intervention appeared to foster a more nuanced and realistic understanding of ageing. Simulation-based approaches may therefore be useful for promoting reflective practice and strengthening awareness of ageing in complex institutional settings.

The use of ageing simulation suits has gained traction in healthcare and caregiving settings as experiential tools designed to simulate some of the sensory, physical, and functional restrictions commonly associated with ageing. Their primary objectives are to foster empathy, challenge ageist attitudes, and promote understanding of older adults and their experiences (Gerhardy et al., 2022). These suits are predominantly used in training programs for health professionals, informal caregivers, and younger demographics (Bowden et al., 2020). However, while interventions with ageing simulation are increasingly established within the healthcare sector *sensu stricto*, its application in other healthcare contexts *sensu lato*, most notably correctional facilities, remains largely unexamined. Yet the global transformation in prison populations brings along new challenges and requirements for the correctional system in catering to an increasingly ageing population.

Amidst global demographic changes and the increasing number of incarcerated individuals of advanced age (see analysis of SPACE I Statistics in Humblet, 2025b), correctional institutions are struggling to navigate more complex and evolving needs. Older people in prison may encounter higher rates of health conditions, disabilities, or functional limitations (Miller et al., 2024; Novisky et al., 2025), which can place significant demands on correctional staff and institutional resources (Williams et al., 2009). Nonetheless, correctional systems have been slow to adapt to the evolving and diverse needs of people in prison as they grow older. While scholars have already advocated for targeted training to address the unique vulnerabilities of ageing prisoners (Cooney & Braggins, 2010; Inglis & Tully, 2016; Wilkinson & Caulfield, 2020), few evidence-based interventions have been developed and empirically tested to date. This contribution seeks to take the first step in closing this gap by discussing the underlying rationale and evaluating the potential benefits of integrating ageing simulation suits into training programs for correctional staff. This line of inquiry thus offers a critical opportunity to assess whether ageing simulation, which is demonstrated to be effective in other domains, can be systematically adapted and meaningfully applied within the training and professional development of correctional staff.

## Background and objectives

The ageing prison population has emerged as a pressing issue in many European countries, including Belgium (Humblet, 2025b). The increasing number of older incarcerated individuals introduces complex challenges for correctional systems, particularly in relation to the provision of adequate care and treatment. Correctional staff operate within an occupational culture shaped by hierarchy, control, and emotional detachment (Farkas & Manning, 1997), a context in which empathy and person-centered care are often undervalued. Previous research indicates that these dynamics can foster systemic neglect of vulnerable populations, including older adults in prison (Crawley & Sparks, 2005; Humblet, 2021). Older adults are especially vulnerable to physical, cognitive, and psychological decline, rendering them at heightened risk of neglect and abuse within the correctional environment (Novisky et al., 2025). Correctional staff have frequently been found to demonstrate limited sensitivity to the needs of ageing people in prison, operating within what Crawley and Sparks (2005) describe as a culture of *institutional thoughtlessness*. This culture reflects a systemic insensitivity that perpetuates ageism both at the organizational level and in everyday staff-prisoner interactions (Humblet, 2021, 2025b).

In response, there is a growing consensus on the necessity of specialized training that equips prison staff with the skills and knowledge required to address the complex health and social care needs associated with carceral ageing (Cooney & Braggins, 2010; Humblet et al., 2021; Inglis & Tully, 2016; Wilkinson & Caulfield, 2020). Yet staff members have limited *embodied understanding* of the sensory and physical constraints inherent to ageing, particularly in such a restrictive context. The absence of experiential insight may not only hinder empathic engagement but could also perpetuate negative, ageist attitudes (Chang et al., 2020; Tremayne et al., 2011) that can be further reinforced by correctional occupational cultures (Humblet, 2025b). While existing educational activities for correctional staff—including training workshops (Masters et al., 2016), computer-based e-learning programs on geriatric and end-of-life care (Loeb et al., 2021), and training modules addressing ageing, mental health, and palliative care in prisons (MenACE, 2018), as well as broader correctional training libraries such as the PO21 project (European Prison Officers for the 21st Century, 2022) and training workshops—provide important cognitive knowledge, they offer little opportunity for direct, experiential engagement.

One promising pedagogical tool can be found in the ageing simulation suit, such as the GERontologic Test suit (hereafter referred to as GERT), which replicates common physical and sensory impairments associated with ageing. Through direct, embodied experiences of age-related limitations, these interventions have demonstrated potential to foster empathy and deepen awareness of the challenges faced by older adults. Empirical research in nursing and healthcare has demonstrated the efficacy of ageing simulation in enhancing caregiver empathy and knowledge. Tremayne et al. (2011) observed that such simulations promote awareness and a more nuanced understanding among healthcare providers and nursing students, respectively. Eymard et al. (2010) further reported that simulation-based training improves both empathy and clinical insight, while Perot et al. (2020) concluded that such sensory interventions increased health professionals’ knowledge and awareness by influencing both their geriatric knowledge and their feelings about age-related limitations. Buys et al. (2023) showed that a “Gray for a Day” simulation, combining theoretical content with experiential exercises, effectively increased participants’ knowledge of age-related functional decline and enhanced their understanding of the challenges faced by older adults. The transformative potential of ageing simulation suits stems from their ability to elicit strong emotional, often visceral, responses that deepen participants’ understanding of ageing challenges (Villaumé et al., 2019). Although initial exposure to these suits may evoke negative emotional reactions or reinforce stereotypical perceptions, due to simulated loss of autonomy and function, these effects tend to be attenuated through guided reflection and group discussion, resulting in improved long-term attitudes toward ageing (Jeong et al., 2017; Jeong & Kwon, 2021; Villaumé et al., 2019).

Surprisingly, the applicability of ageing simulation has also been explored in a particular law enforcement context. A U.S.-based study employing an *‘Aging Simulation Sensitivity Training Kit’* reported that police officers experienced increased empathy and adapted communication strategies, such as speaking more clearly and with heightened awareness of physical limitations, when interacting with older adults (Brown et al., 2017). Given the parallels between policing and corrections, including hierarchical structures, rule-oriented cultures, and emotionally demanding, risk-laden environments that are predominantly occupied by males (Farkas & Manning, 1997), there is reason to believe that ageing simulation interventions could be similarly effective within correctional settings. Both roles are shaped by norms that may resist emotional openness or empathetic engagement, especially when directed toward marginalized populations such as justice-involved older adults.

Despite promising findings in healthcare and policing, the application of ageing simulation suits in correctional staff training remains largely unexplored. To date, the effectiveness of ageing simulation suit interventions in correctional geriatric education has not yet been systematically or robustly evaluated, underscoring the need for exploratory research in this area. This gap highlights the importance of investigating whether ageing simulation can meaningfully enhance staff awareness, knowledge and attitudes in responding to the needs of incarcerated older adults, thereby contributing to a cultural shift toward more respectful and responsive geriatric care in prisons. To address this gap, the present study builds on evidence from related fields demonstrating the potential of ageing simulation to enhance professional attitudes and care practices (Perot et al., 2020; Villaumé et al., 2019). As an exploratory pilot study, this study aims to generate initial insights into whether a correctional geriatric care training incorporating ageing simulation could enhance understanding of and potentially shape attitudes toward ageing among correctional officers. To this end, we designed, implemented, and preliminarily evaluated the first experiential ageing simulation suit-based intervention embedded within a Belgian geriatric care training program for correctional staff.

## Research design and methods

This study piloted an ageing simulation intervention within a newly developed geriatric care training module for correctional officers in Belgium. The study integrated the GERT Age Simulation Suit (GERT suit) into a novel prison officer training module. See Supplementary Text 1 for details on the module and the GERT simulation suit.

The intervention combined a theoretical module, building on earlier work (Humblet et al., 2021), with a practical simulation using the GERT suit. It was designed to provide officers with embodied insights into physical, auditory, motor, and postural limitations, thereby offering officers embodied insights into (some of) the challenges experienced by incarcerated older people. During the simulation exercises, participants could experience three possible roles: (a) wearing the ageing simulation suit and acting as an older prisoner, experiencing (some of) the physical and sensory constraints associated with ageing, (b) interacting with the suited participant in the role of correctional officer, or (c) observing and reflecting on the interaction. While all participants had the opportunity to observe the simulation scenarios, wearing the ageing suit itself was voluntary, and not all participants chose to take on this role. The GERT suit was used to replicate impairments in vision, hearing, mobility, dexterity, and posture, thereby facilitating embodied learning experiences. The training involved a 4-hr simulation session, including pre-briefing, role-playing scenarios, and debriefing discussions. Scenarios were grounded in previous research (Humblet, 2021) and developed in collaboration with experienced trainers (working at the educational center for correctional staff) to reflect typical daily activities of older adults in correctional facilities (e.g., communicating, mobility, getting dressed, climbing stairs, eating, cleaning cells, following instructions) (see Supplementary Text 1 for the scenarios). The scenarios were pilot-tested by the trainers in a separate session to ensure feasibility and realism. Each scenario, lasting around 5 min, provided participants with an embodied experience of some of the sensory and functional restrictions associated with normal ageing, as simulated by the suit. A mixed-methods research design was employed to assess cognitive, affective, and attitudinal outcomes.

### Recruitment

Nineteen correctional officers were recruited from Dutch-speaking prisons in the northern part of Belgium with a high concentration of older adults, primarily through self-selection. In one facility, however, participation of staff working on a unit for care-dependent older adults was encouraged by the prison governor. Both the researcher and the gatekeeper (training center contact person) explicitly emphasized that participation was entirely voluntary. As a result, two participants, both holding senior hierarchical positions, withdrew prior to the intervention. An additional participant was unable to attend due to illness. During the intervention, participants rotated through the roles of incarcerated older adults, correctional officers, or observers.

### Study design and data collection

**Quantitative data** were collected through pre- and post-intervention surveys adapted from validated instruments (see Supplementary Text 1 on the instrument adaptation process):

- Attitudes toward older adults were measured using selected items from the *Kogan Attitudes Toward Old People Scale* (Kogan, 1961). Items were rated on a Likert scale (1 = strongly disagree to 5 = strongly agree). Negatively worded items were reverse-scored so that higher scores indicate more positive attitudes. Items were analyzed at the individual level (focusing on a subset of the most relevant items (see Supplementary Table 1; cfr. infra limitations section).
- Attitudes toward working with older adults were measured using items adapted from the *Willingness to Work with Elderly People Scale* (Momtaz et al., 2018). Items were rated on a Likert scale, and mean scores were calculated (after reverse scoring where applicable), with higher scores indicating greater willingness, more positive attitudes, and higher perceived competence.
- Age-related prejudice was assessed using the *Ambivalent Ageism Scale* (Cary et al., 2017), consisting of benevolent (9 items) and hostile (4 items) subscales. Items were rated on a Likert scale, and mean scores were calculated per subscale. Higher scores indicate stronger endorsement of benevolent or hostile ageism. No reverse scoring was applied.

The survey included demographics and additional knowledge-based items adapted from a prior e-course on (the needs of people) ageing in prison (Humblet et al., 2021). Knowledge was assessed using 9 items covering key domains relevant to ageing in detention (e.g., physical ageing, cognitive decline, dementia, stroke recognition, and sensory impairments). Each item was coded as correct (1) or incorrect (0) and summed to create a total knowledge score ranging from 0 to 9, with higher scores indicating greater knowledge.

The survey also included open-ended reflection prompts, such as

- *What did you find valuable about this training?*
- *What could be improved?*
- *What is something you learned today?*
- *What do you plan to do differently in your work as a result of this training?*
- *Is there anything you would like to add?*

### Analyses

Quantitative analyses were primarily descriptive and exploratory. Pre-post differences were examined using paired-sample *t*-tests to assess within-person change in knowledge, attitudes toward older adults, willingness to work with this population, and ageism. Given the exploratory nature of the study and the small sample size (*n* = 19), the results were interpreted cautiously as preliminary indications of possible patterns rather than confirmatory evidence. Because the study involved repeated measures within the same participants, paired tests were considered appropriate for assessing within-person change. Results from subgroup analyses, including those based on whether participants wore the suit, were considered especially exploratory. Some participants did not complete all questionnaire items or were absent at post-test, resulting in missing data. Paired analyses were conducted using listwise deletion, including only participants with complete pre- and post-test data for each measure. Consequently, the sample size varied slightly across analyses. A *p*-value below .05 was considered statistically significant, indicating that the observed results were unlikely to be due to chance and thus provide preliminary evidence of intervention effectiveness.

The study relied on existing English scales that had not been specifically developed or previously validated for this target group or research context. Although a forward-backward translation procedure was applied and face and content validity were checked, further research should investigate internal and external validity. This could be achieved through larger sample sizes and testing the reliability of the scales across different contexts and populations.

**Qualitative data** were collected through two post-intervention focus group discussions designed to explore participants’ emotional responses, reflections, and perceived changes (see Supplementary Text 2 for the focus group discussion guide). These narratives offered qualitative indicators of learning outcomes, emotional engagement, and unexpected effects. Each focus group (lasting approximately 1 hr) involved correctional officers who had participated in the training and role-play exercises. Participants had the option to wear the GERT suit, observe its use, or act as an officer within the simulation. Discussions focused on participants’ experiences, perceptions, and potential shifts in attitudes or practices following the intervention. All sessions were audio-recorded and transcribed verbatim for analysis. Thematic analysis followed Braun and Clarke’s (2024, 2006) six-step framework. The analysis was primarily inductive but was also guided deductively by the study objectives, with particular attention to participants’ physical and emotional reactions to the ageing suit, its influence on empathy and understanding, its relevance to daily practice, and any reported changes in attitudes or work behaviors. Familiarization, coding, and theme development were conducted separately for each focus group before being compared and integrated. Final themes were defined, described, and illustrated with representative quotations, capturing both positive reflections and critical feedback, including suggestions for improvement.

## Results

### Participants

A total of 19 participants (16 men; 3 women) took part in both pre- and post-intervention assessments and focus groups. See Supplementary Table 2 for the participant characteristics. The first focus group consisted of all men (*n* = 9), whereas the second consisted of 3 female and 8 male participants (1 male participant did not take part in the post-intervention measurement). They were employed at Merksplas (*n* = 9), Brugge (*n* = 7), Dendermonde (*n* = 2), and Oudenaarde (*n* = 1), with most working in specialized units for older inmates (*n* = 16). Thirteen participants had prior experience working with older people in prison, while 12 had previous professional experience with older people, and 9 had personal experience with older adult care. In addition to 17 correctional officers, a team leader and a detention counselor participated. Participants’ ages ranged from 25 to 64 years, with work experience ranging from 1–5 years (*n* = 3) to over 20 years (*n* = 2). The majority (68%, *n* = 13) had 11–20 years of experience in prison settings. Regarding education, 58% (*n* = 11) had completed secondary school as their highest qualification. Nine participants had previously attended training on care for older people in detention, primarily through the Oscar e-learning program (https://ecourses.odisee.be/icto/cursus/oscar/). Participation in wearing the simulation suit varied across sessions; see Supplementary Table 3 for detailed information on role assignments, suit usage, and observations for each session. In the first session, two participants wore the suit during the scenarios. In the second session, the majority of participants engaged with the suit at least partially, although some chose not to wear it during the active scenarios. Overall, nine of the nineteen participants wore (parts of) the suit at some point. Participants generally reported feeling more comfortable trying the suit between scenarios rather than during them. An unexpected but meaningful finding was participants’ reluctance to wear the ageing simulation suit. For some, the exercise involved confronting their own ageing, or that of significant others, and imagining future physical or cognitive decline. For others, role conflict appeared to arise when taking the perspective of a prisoner conflicted with professional identity and norms of authority, control, and emotional restraint. Participation was also shaped by group dynamics: skepticism among peers appeared to reinforce hesitation in one group, whereas reflective peer engagement in the other group seemed to make participation feel more legitimate and psychologically safe. This suggests that uptake of simulation-based interventions depends not only on individual motivation but also on occupational culture and the social conditions under which training is delivered.

### Quantitative results

Knowledge scores improved modestly (mean increase from 6.42 to 7.16), nearing statistical significance (*p* = .055). Attitudinal shifts were nuanced: while general attitudes toward incarcerated older adults did not significantly improve, participants who wore the ageing simulation suit showed increases in willingness and perceived competence in working with older prisoners (*p* < .05). These differences reached statistical significance within this small subgroup, but given the limited number of participants who wore the suit, these findings should be interpreted as exploratory. Ageism measures remained fairly stable. See Supplementary Table 4 for the full results.

#### Knowledge

Participants showed a small but positive increase in knowledge of ageing-related issues. Participants showed increased understanding of physical limitations (reduced muscle strength, mobility, sensory impairments), functional challenges (daily activities), and social/cognitive consequences (isolation, language difficulties). However, statistical significance was not reached (*p* = .055), suggesting the need for larger sample sizes in future research. Of the nine knowledge questions, participants scored an average of 6.42 (standard deviation = 1.43) in the pretest and 7.16 (1.26) in the post-test, showing a modest increase, though this did not reach statistical significance (*t*(18) = −1.68, *p* = .055). A larger sample is needed to confirm whether the intervention effectively enhances knowledge of ageing. On individual questions, most showed improvement, except for 1 vignette related to mental/cognitive ageing, where fewer participants answered correctly in post-intervention. This may reflect increased awareness of the complexity of cognitive changes in older adults, leading participants to consider multiple possible explanations rather than selecting a single answer. Alternatively, this finding may reflect variability associated with the small sample size and the interpretative nature of vignette-based questions.

#### Attitudes

Regarding attitudes toward older adults, the intervention did not produce the expected patterns. Due to low internal consistency, attitudes were analyzed at the item level (Supplementary Table 1). Overall, changes were small and largely nonsignificant. Participants showed slightly stronger agreement with the statement that older adults should be housed in separate units (*M*_pre_ = 4.21 [1.36]; *M*_post_ = 4.47 [1.12]). Those who wore the ageing suit were initially more supportive and slightly softened their stance post-intervention. Beliefs about the adaptability of older adults showed mixed patterns, with slightly weaker agreement that older adults are “set in their ways” (*M*_pre_ = 4.74 [0.87]; *M*_post_ = 4.42 [0.61] but also slightly lower agreement that older adults can easily adapt to changing situations (*M*_pre_ = 3.26 [1.05]; *M*_post_ = 3.00 [1.00]). Perceptions of wisdom became more ambivalent, with agreement increasing for both the idea that wisdom does not necessarily come with age (*M*_pre_ = 3.00 [1.25]; *M*_post_ = 3.58 [1.12]) and that people become wiser over time (*M*_pre_ = 3.47 [1.22]; *M*_post_ = 4.05 [1.23]). Participants may have become more aware of the complex relationship between age and wisdom. Most other items showed minimal change, including perceptions of social interaction and diversity among older adults. One item, regarding whether older adults appear clean and well-presented, showed a significant decrease (*M*_pre_ = 4.32 [0.82]; *M*_post_ = 3.95 [0.85]; t(18) = 2.35, *p* = .015).

Overall, the results suggest that attitudes did not become more positive, but rather more nuanced and, in some cases, more critical. The intervention did not lead to unequivocally more positive attitudes, but instead prompted a more nuanced view of ageing, acknowledging both the limitations and potential for change.

#### Willingness to work with older people and competency

At the group level, no significant changes were observed in attitudes, perceived competence, or motivation. Mean scores showed slight increases across dimensions (e.g., attitudes: *M*_pre_ = 5.23 [0.92]; *M*_post_ = 5.36 [0.73]; competence: *M*_pre_ = 3.92 [0.85]; *M*_post_ = 4.04 [1.00]), but these differences were not statistically significant. Among participants who wore the ageing simulation suit, significant increases were observed in attitudes and perceived competence to work with older adults (in prison) (*t*(5) = −2.89, *p* = .017; *t*(5) = −2.13, *p* = .043), while motivation remained unchanged. Given the very small subgroup size, these findings should be interpreted as exploratory.

#### Ageism

Exploratory patterns suggest that age-related biases (benevolent and hostile ageist attitudes) remained relatively stable overall. Among participants who wore the ageing suit, slight increases in hostile ageism were observed. However, due to the small subset, these changes should be interpreted with caution and may reflect a heightened awareness of the physical and psychosocial challenges associated with ageing.

#### Participant evaluation

Participants reported learning valuable lessons from the intervention, with many recognizing the physical and mental challenges of ageing. Many participants expressed a desire to show more patience and empathy toward older inmates and become more aware of their social needs. Positive feedback indicated that the mix of theory and practice was appreciated, but some participants suggested incorporating more situational exercises and offering greater flexibility regarding who would wear the ageing suit, which components would be used, and how long participants would engage with the simulation. Participants also recommended integrating practical components more closely with theoretical content rather than structuring the program across two separate half-day sessions.

### Qualitative results

Thematic analysis of the two focus groups revealed five interconnected themes reflecting the interplay between empathy, professional identity, institutional constraints, relational dynamics, and the perceived value of the intervention. Participants’ reflections demonstrated both transformative learning and resistance, shaped by organizational culture and role expectations. The analysis was guided by complementary theoretical perspectives (see Table 1 below for an overview of the themes and analytical levels). Firstly, transformative and experiential learning theories (Kolb, 1984; Mezirow, 1997) informed us on how embodied experiences may foster perspective-taking and reflexive understanding. Secondly, scholarship on emotional labor (Hochschild, 1983; Humblet, 2020) provided some insight into professional norms in correctional work, highlighting how officers regulate emotions, maintain professional distance, and use coping strategies in response to challenging interactions. Thirdly, research on institutional logics and care-custody tensions demonstrates how organizational rules and structural constraints might prevent officers from acting according to their perceived moral responsibilities, giving rise to moral distress (Jameton, 1984). Finally, work on ageism and carceral ageing (Humblet, 2025b; McLaren et al., 2025) offered a lens for understanding relational tensions and perceptions of “older prisoners”.

**Table 1:** Overview of themes and analytical levels.

Level	Theory	Themes
**Individual**	Transformative learning	Feeling ageing
**Professional**	Emotional labor	Professional distance, emotion regulation
**Institutional**	Moral distress	Institutional tensions
**Social perception**	Ageism	Relational tensions

#### Feeling ageing: embodied empathy and reflexive understanding

The focus groups provided insights into both the physical and psychological experiences of participants who wore or observed others wearing the ageing suit. This experience was both physically and mentally confronting, yet highly educational. Participants experienced limited mobility, sensory impairments (such as reduced sight and hearing), diminished fine motor skills, and physical exhaustion. The most frequently reported physical issues included exhaustion, stiffness, slowness of movement, and difficulty with simple tasks like standing or walking: “You really have to put effort into getting up” or “Walking properly and all the steps… yeah, that was hard”. The sensory impairments, notably the glasses and headphones, reduced sight and hearing to the point where participants described feeling disoriented “It felt like I was swaying” and “I began to doubt my senses” to feeling socially isolated from their environment “I could barely hear anything.” Furthermore, diminished fine motor skills made everyday tasks, such as writing or spreading butter, feel unnatural and laborious. Some participants even noted that the experience was physically exhausting despite the brief duration of the suit’s use: “If you wore this for an hour, you’d really feel it.”

The physical discomfort was accompanied by strong emotional and psychological responses. The most common reaction was an increased sense of empathy and understanding for older individuals: “I realize that now, by carrying it myself… I have a bit more understanding” (Speaker 13, Group 2); “Once you experience it yourself, you understand why everything slows down a bit.” (Speaker 2, Group 2).

Most participants reported that the suit helped them empathize more with the challenges faced by older people, particularly regarding the slowness or difficulty in everyday tasks. Some expressed how the experience confronted them with feelings of frustration, powerlessness, and social isolation, especially since they were unable to engage fully with their surroundings (e.g., conversations): “You’re actually imprisoned in yourself” (Speaker 10, Group 2).

The feeling of being imprisoned or feeling trapped inside your own body mirrors the experiences of older adults in prison as described in previous research (Humblet, 2021; Humblet & Decorte, 2013). This understanding also extended to how ageing manifests within the correctional context, where mobility is restricted, sensory stimuli are minimal, and assistance is not readily available: “When you’re in prison and you’re also old… yeah, everything becomes twice as hard.” (Speaker 3, Group 2).

Thus, the simulation made the abstract realities of ageing more tangible, promoting a deeper awareness of the challenges of carceral ageing. This aspect of the training was seen as crucial for fostering empathy, challenging preconceived notions, and encouraging more tailored activities and care practices.

These comments illustrate what Mezirow (1991) terms *transformative learning*, i.e., a shift in perspective prompted by a disorienting dilemma and self-reflection. Participants described a visceral awareness of physical decline and vulnerability, translating abstract knowledge about ageing into embodied understanding that goes beyond cognition. This process aligns with notions of *embodied empathy* and *experiential learning*, where physical immersion fosters emotional insight and perspective-taking. While the simulation increased understanding, it sometimes evoked feelings of helplessness and highlighted the discomforts of ageing, rather than its strengths. These insights echo previous findings (Villaumé et al., 2019) that realism may initially provoke anxiety, but lead to longer-term empathy gains. The simulation prompted a more realistic, though not necessarily more optimistic, view of ageing.

#### Not feeling aging: professional identity and distance

Not all participants embraced the experiential component. Several prison officers resisted wearing the ageing suit or engaging in role-play, citing discomfort or skepticism about its relevance:

“I’ve already worked a lot in this field. I’m empathetic enough to deal with these men. I know how to handle it.” (Speaker 7, Group 1)

“I’ll just sit until five minutes to three. I’m not doing it!” (Speaker 9, Group 1)

“We already see enough misery.” (Speaker 9, Group 1)

Such resistance reflects underlying tensions between *professional identity* and *experiential vulnerability*. In a correctional culture where emotional control and detachment are associated with competence, engaging in role-play or visibly struggling with the suit challenged participants’ self-perception as competent professionals. The lack of volunteers for wearing the ageing suit suggested some level of hesitation, discomfort, or concern about visibility or taking a central role. One participant stated simply, “I don’t like being in the spotlight.” (Speaker 7, Group 1). These patterns align with the literature on emotional labor (Hochschild, 1983) and research on emotions in organizations (Fineman, 2008), which highlights how professional norms can discourage the open display of vulnerability or emotion. Experiences that make participants emotionally or experientially vulnerable can provoke resistance, in the form of emotional pushback, hesitation, or defensiveness, particularly among professionals accustomed to authority and composure.

Differences between groups were notable. The first group relied on (cynical) humor and irony to diffuse discomfort, while the second group adopted a more reflective tone, occasionally critiquing (management) practices. These variations underscore that experiential learning outcomes are shaped not only by content but also by group climate and institutional culture. The second group engaged more in reflection, including subtle criticism of management. Group humor served here as a collective coping mechanism: “That’s funny, isn’t it, colleagues, seeing [this]?” (Speaker 4, Group 2).

Humor and irony can signal both solidarity and resistance, reflecting an organizational culture where emotional distance is normalized. The group interactions suggested that learning outcomes were shaped not only by content but by the social climate of the professional group. Effective experiential learning requires psychological safety and sensitivity to professional hierarchies. These additional observations reflect differences in the learning environment and group climate, influenced by factors such as age, gender, role, and work context.

#### Institutional tensions: safety, rules, and moral ambiguity

The balance between safety, rules, and professional conduct refers to the constant tension that prison staff must navigate between ensuring safety and following regulations while fulfilling their professional duties. This highlights the challenges of working in an environment where strict rules and safety protocols sometimes conflict with the need for human intervention.

Across both focus groups, participants articulated a persistent tension between procedural safety, regulatory compliance, and humane care. The statement “We do this, but, yeah, it’s not supposed to be done. But if someone is lying there on the floor, what do you do?” (Speaker 2, Group 1) captures the moral uncertainty surrounding physical assistance to “prisoners”. Another correctional officer voiced concern about potential consequences: “You’d want to be sure… What if I help them up, and their hip is broken, and my action makes things worse?” (Speaker 9, Group 1).

Many participants considered themselves empathetic, but the hesitation to act seemed to stem from uncertainty about their roles and the potential repercussions of their actions. The need for clear, written guidelines and protocols was expressed: “Our action plans focus more on behavior and aggression, but not on physical assistance or these situations.” (Speaker 10, Group 1).

Both focus group discussions highlighted concerns regarding the psychological and physical strain of their roles. Stress, role uncertainty, and fears about physical safety were recurring themes, alongside concerns about time pressure and the balance between maintaining security and respecting privacy: “It’s like when someone is in the shower. We don’t stand by them either.” (Speaker 6, Group 1)

Such stressors not only affect staff well-being but also the quality of care they can provide. Fatigue, stress, and anxiety can lead to errors, reduced empathy, and a negative attitude toward inhabitants. Against this background, the demand for greater support in terms of more staff was expressed.

“When we call the nurses and they say ‘leave them there’ what do you do?” (Speaker 9, Group 1)

These dilemmas exemplify moral distress (Jameton, 1984), i.e., knowing the right action but being constrained by institutional policy or fear of liability. Participants often invoked the phrase “It’s not my job”, revealing how procedural rigidity and ambiguous responsibilities suppress compassionate responses. The absence of well-defined protocols and the lack of clear division of responsibilities generated persistent frustration and uncertainty. This, in turn, imposed a hidden burden of emotional labor, as staff had to navigate moral tension, manage their own emotional responses, and negotiate with healthcare personnel under ambiguous role conditions, echoing Humblet’s (2020) argument about how unclear institutional expectations amplify emotional work.

These accounts underscore that empathy and ethical conduct are constrained not only by individual attitudes but also by structural and procedural ambiguity and limited accountability.

#### Relational tensions: empathy, frustration, and moral judgment

Participants demonstrated varied and sometimes contradictory attitudes, ranging from concern and helpfulness to frustration and even punitive views. They expressed ambivalent attitudes toward aging prisoners, oscillating between empathy and irritation. Some described their mutual respect and adaptive behavior.

Some participants expressed concern and a willingness to help older inhabitants, especially in case of medical emergencies. Against this background, it was noted that incarcerated individuals at times showed respect for older peers, such as: “They even give up their bed to let the older detainee sleep on the lower bunk” (Speaker 5, Group 1).

This demonstrated an awareness of adjusting behavior to meet the needs of older people in corrections. However, not all staff were equally motivated to assist older adults, especially those who were less mobile or exhibited passive behavior: “If they take longer to get dressed, it’s not like you should stay with them” (Speaker 7, Group 1).

Others expressed frustration with older adults perceived as slow or passive: “When they slump down, you have to drag them up, and that’s frustrating” (Speaker 2, Group 1).

Some participants showed indifference toward detainees displaying signs of social withdrawal, interpreting it as laziness: “They become lazy. They don’t even get out of bed to get their food” (Speaker 2, Group 2).

This may also reflect forms of ageism within correctional environments. Ageist perceptions often frame older individuals as frail, dependent, or burdensome, which can shape staff expectations and responses to older adults. Research on carceral aging similarly highlights how older incarcerated individuals may be perceived simultaneously as vulnerable and as undeserving of care, particularly when their offenses are considered morally reprehensible (e.g., Humblet, 2021; 2025b; McLaren et al., 2025).

Biases based on the crimes committed by older incarcerated individuals seemed to create a barrier to empathy. Empathy was described as conditional, shaped by perceptions of the offense and the perceived deservingness of the prisoner. This was especially linked to individuals incarcerated for sexual offenses, as having committed particularly heinous crimes: “I can’t even acknowledge them; it’s hard to care for someone like that” (Speaker 8, Group 1).

Despite these challenges, respect for older prison inhabitants was generally observed, as reiterated by one of the participants: “It works both ways. If you respect them, they’ll respect you too” (Speaker 14, Group 2).

These ambivalences reflect the moral complexity of care within custodial settings. Discussions revealed deeper professional and institutional tensions. Staff reflected on the challenge of balancing safety, rules, and care when assisting older adults with physical needs, consistent with research showing that correctional staff not only negotiate custodial duties and caring roles but also experience emotional labor and care-security role conflicts in their work with vulnerable groups (Humblet, 2020; Sundt et al., 2024). Empathy toward aging individuals, although achievable, emerged in the focus groups as both an individual emotional response and a practice constrained by institutional norms and moral judgment.

#### Learning and reflection in the aging simulation

This last theme covers participants’ experiences of the ageing suit intervention, highlighting the nuanced ways in which it aligned -or failed to align- with their professional perspectives and learning needs. Reactions to the aging simulation training were mixed. Some participants expressed reluctance toward role-playing, reflecting discomfort or skepticism about its relevance. Some participants questioned the added value of the simulation rather than engaging with, and reflecting on, the experience. Conversely, for others, the embodied experience proved transformative, with initial apprehension giving way to reflection and appreciation: “At first, I was scared… but I’m glad I wore it. I’m really happy I did” (Speaker 6, Group 2).

Many participants valued the shift between theory and practice, noting: “It was nice to have a break from the theory” (Speaker 2, Group 2).

Participants also reflected on the relevance of the intervention across different staff groups. Some recognized its potential benefits for younger or less experienced colleagues, while others questioned its necessity for those with prior experience in care settings, illustrating resistance (see supra): “I think we already have this, because I have a care background, so I’ve studied this. I already see things through that lens” (Speaker 12, Group 2).

The retention and perceived impact of the training varied. While some participants worried that insights would fade quickly: “I think it will stick for a while, but then it will fade. You’ll remember some of it for a week, then within two weeks, it’s like, ‘What was that again?’” (Speaker 10, Group 2). Others were more optimistic about its long-term effects, asserting: “It will stick, it definitely will” (Speaker 9, Group 2).

Overall, the focus group findings suggest that the ageing simulation successfully elicited understanding, empathy, and reflection, while also exposing professional defensiveness and structural barriers. The intervention highlighted tensions between custodial control and care ethics, indicating that sustainable change requires not only experiential insight but also organizational support and systemic reform.

### Limitations

The quantitative study’s small sample size (*n* = 19) limits its statistical power and generalizability. The limited sample size may have reduced statistical power to detect small or moderate changes. Although the sample size was small, the study focused on a highly specialized group of correctional officers working in dedicated units for older prisoners. Given that only two such units exist in the region, the participants represent a substantial portion of the relevant professional population.

A one-tailed test was used to focus on detecting positive effects in line with the hypothesis that the intervention would enhance knowledge and attitudes toward older adults. While *p*-values below .05 are generally interpreted as indicating a low likelihood that results are due to chance, these findings should be interpreted cautiously given the limited sample. The absence of a control group further restricts the ability to attribute observed changes solely to the intervention. Without random assignment, other unmeasured factors (such as prior experiences, workplace culture, or social desirability in self-reported responses) may have influenced participants’ answers.

Existing English-language scales were adapted, though they were not originally developed for, or validated, in this specific context. Internal consistency of the Kogan Attitudes Toward Old People Scale was low in the present sample, precluding the use of a reliable composite score (see Supplementary Table 1). This may reflect a mismatch between the scale and the correctional setting, the limited sample size, and the known sensitivity of the Kogan scale to translation and contextual variation (e.g., Iwasaki & Jones, 2008). Although forward–backward translation and assessments of face and content validity were conducted, further research is needed to examine the reliability and construct validity of these instruments among correctional staff. Future studies should aim for larger, more diverse samples, include longitudinal follow-up, and employ context-specific and psychometrically robust measures to enhance the credibility and generalizability of findings.

Overall, the intervention did not lead to clearly more positive attitudes toward older prisoners but seems to have led to a more realistic and nuanced understanding of ageing. The specific role participants took in the intervention may have influenced the outcome, though the small sample size prevented systematic testing. Participants who had worn the aging simulation suit showed indications of improved attitude and care competency, suggesting that direct experience of ageing can positively shape perceptions of working with older individuals. As only a subset of participants wore the suit, these findings are exploratory and should be interpreted with caution. Moreover, participation was voluntary and primarily based on self-selection; some staff members opted not to participate, which may have introduced selection bias and influenced both the quantitative outcomes and the group dynamics observed during the qualitative study. Two candidates, both holding senior hierarchical positions, withdrew prior to the intervention. This may have further affected participation rates and group discussion dynamics. Future interventions may therefore require additional efforts to boost motivation and engagement, such as incorporating role-playing scenarios with rewarding effects, like receiving gratitude from “older adults” or positive interactions (compliments, smiles) in response to respectful and attentive behavior. In addition to that, as outcomes were measured immediately after the intervention, it remains unclear whether observed improvements were maintained over time. Approximately half of the participants had previously attended an online learning on aging in prison. This prior exposure may have influenced baseline knowledge or attitudes and may partially explain the limited changes observed in some outcomes. The participant group consisted predominantly of men, reflecting the gender composition of correctional staff. Gendered occupational norms in correctional environments may influence emotional expression and engagement with experiential learning exercises, and future research should explore these dynamics further.

Despite these limitations, the study provides valuable preliminary insights for designing and evaluating similar interventions in correctional environments.

## Discussion and implications

Taken together, the mixed-methods findings from this pilot study suggest that, while measurable changes in attitudes were limited, the experiential nature of the ageing simulation prompted meaningful reflection among participants. Although the quantitative results did not demonstrate clear attitudinal shifts, the qualitative data revealed reflections related to empathy, patience, and increased awareness of aging-related challenges. This indicates that the intervention may primarily foster reflection and perspective-taking, which could precede measurable changes in attitudes.

From a theoretical perspective, interventions that facilitate embodied learning, i.e., where knowledge is acquired through bodily and sensory experience, offer a promising pedagogical pathway. Drawing on traditions of experiential learning (Kolb, 1984) and empathy training (Riess, 2017), ageing simulation suits make age-related impairments tangible by simulating limitations in vision, hearing, mobility, and dexterity. This exposure challenges abstract or stereotypical notions of older adults while encouraging reflexivity about institutional factors that may exacerbate age-related vulnerabilities. As such, ageing simulation suits serve not only as practical training tools but also as theoretically grounded interventions with transformative potential in reshaping professional practice and challenging bias. This study contributes to research on carceral ageing by demonstrating that experiential training interventions rather function as reflexive encounters that expose underlying professional norms and institutional constraints, generating, potentially, both empathic understanding and defensive resistance toward ageing people in prison. The aging simulation should not be viewed as an end goal in itself, but as a starting point for reflexive learning. By creating embodied experiences of ageing, the tool exposes underlying professional norms, emotional labor demands, and institutional constraints, prompting staff to reflect on both their attitudes and practices toward older prisoners. Its value lies in opening space for discussion, reflection, and organizational change, rather than simply generating an outcome.

Against this background, it should be noted that experiential ageing simulation in corrections training represents both a conceptual and a cultural intervention. It exposes staff members to vulnerabilities that are often downplayed or overlooked in correctional culture, where strength and control are dominant norms. The intervention may bring about an increased realism, initially reducing perceived competence or, in some cases, reinforce ageist attitudes. A nuanced approach is therefore required, where simulation serves as an entry point for sustained professional development rather than a one-off experience. When paired with dialogue and theoretical reflection, these simulations can foster deeper professional awareness and intended responsiveness. Moreover, the findings show that changes among participants were not uniform: while some reported negative associations or reinforced stereotypes, most reported enhanced understanding and increased patience. The identified barriers included discomfort with role-play, limited exposure to the simulation, and preexisting skepticism, whereas the facilitators comprised open discussion, skilled trainers, and peer participation. Both individual-level barriers and the broader occupational culture are important considerations for future implementation. Reflection sessions have consolidated learning, while role assignment and the broader institutional context mediated effectiveness. The study seems to suggest that similar simulation-based interventions should be designed around small, trust-based settings that encourage shared reflection. The long-term impact of ageing simulation interventions on professional behavior and institutional practice in corrections remains to be established. While the present findings provide preliminary support for the value of experiential ageing simulation, further research is needed using larger samples, comparative study designs, and longitudinal follow-up to determine whether such interventions lead to sustained changes in correctional officers’ attitudes and practices toward older prisoners. Future iterations may also benefit from embedding ageing simulation within a broader curriculum addressing diversity, care ethics, and age-sensitive practice.

## Conclusion

Although widely employed in healthcare and caregiving contexts (Gerhardy et al., 2022), the use of ageing simulation suits within correctional settings remains largely underexplored. This study highlights a significant gap and considerable untapped potential for extending ageing simulation research to other professional groups working in under examined environments where older adults reside or interact, such as prisons, jails, police stations, and psychiatric facilities.

This study has offered valuable insights into whether and how ageing simulation can serve as an effective tool for training correctional staff. Although the intervention did not universally result in more positive views toward older incarcerated adults, it did foster critical reflection and enhanced awareness of the needs and challenges faced by aging individuals in correctional settings. By momentarily disrupting patterns of thoughtlessness (Crawley, 2005), it encouraged correctional staff who wore the suit to reconsider and adapt their relational practices with aging individuals. These outcomes align with *institutional thoughtfulness* (Humblet, 2021), reflecting a shift toward greater awareness of, and responsiveness to, the needs of individuals growing older in corrections. However, it remains important for such efforts to be reinforced and supported through institutional policy.

Future research should further examine the potential of both in-person and virtual simulations to enhance correctional staff’s understanding of, and responsiveness to, the needs of an increasingly ageing prison demographic. Longitudinal studies, studies with larger and more diverse samples, with extended and repeated exposure, and/or the inclusion of control groups are warranted. Additionally, the potential of ageing simulation training for peer caregivers within prisons -a currently underexplored yet promising area- should be examined (Du Toit et al., 2019; Loeb et al., 2021). There is a clear need to establish an empirical evidence base evaluating the efficacy of ageing simulation suit interventions in correctional settings. This study represents a first step toward that goal, aiming not only to inform corrections training practices but also to advance broader discussions on ageing and institutional responsibility within correctional settings.

## Supplementary material

Supplementary material is available at *Innovation in Aging* online.

## Supplementary Material

igag075_Supplementary_Data

## Data Availability

The study was approved by the ECHS (The Ethical Committee for Human Sciences) at the Vrije Universiteit Brussel [ECHW_334]. The full report is publicly available through the institutional repository of the Vrije Universiteit Brussel (see Humblet, 2025a). As complete anonymization could not be fully guaranteed, the data are not publicly available.

## References

[igag075-B1] Bowden A. , Wilson V., Traynor V., Chang H.-C. R. (2020). Exploring the use of ageing simulation to enable nurses to gain insight into what it is like to be an older person. Journal of Clinical Nursing, 29, 4561–4572. 10.1111/jocn.15484 32890451

[igag075-B2] Braun V. , Clarke V. (2006). Using thematic analysis in psychology. Qualitative Research in Psychology, 3, 77–101. 10.1191/1478088706qp063oa

[igag075-B3] Braun V. , Clarke V. (2024). Thematic analysis. In Maggino F. (Ed.), Encyclopedia of quality of life and well-being research (pp. 7187–7193). Springer International Publishing.

[igag075-B4] Brown R. , Ahalt C., Rivera J., Stijacic C. I., Wilhelm A., Williams B. (2017). Good cop, better cop: Evaluation of a geriatrics training program for police. Journal of the American Geriatrics Society, 65, 1–6. 10.1111/jgs.14899 PMC5555774 28436006

[igag075-B5] Buys D. , Harris-Speight J., Redwine P., Sansing A., Yelland E. (2023). Gray for a day: A simulation activity impacts participants in community-based health education. Innovation in Aging, 7, 839. 10.1093/geroni/igad104.2705

[igag075-B6] Cary L. A. , Chasteen A. L., Remedios J. (2017). The Ambivalent Ageism Scale: Developing and validating a scale to measure benevolent and hostile ageism. The Gerontologist, 57, e27–e36. 10.1093/geront/gnw118 27520730

[igag075-B7] Chang E.-S. , Kannoth S., Levy S., Wang S.-Y., Lee J. E., Levy B. R. (2020). Global reach of ageism on older persons’ health: A systematic review. PLoS One, 15, e0220857. 10.1371/journal.pone.0220857 31940338 PMC6961830

[igag075-B8] Cooney F. , Braggins J. (2010, December 7). Doing time: Good practice with older people in prison–the views of prison staff. Prison Reform Trust. https://prisonreformtrust.org.uk/doing-time-good-practice-with-older-people-in-prison-the-views-of-prison-staff/

[igag075-B9] Crawley E. (2005). Institutional thoughtlessness in prisons and its impacts on the day-to-day prison lives of elderly men. Journal of Contemporary Criminal Justice, 21, 350–363. 10.1177/1043986205282018

[igag075-B10] Crawley E. , Sparks R. (2005). Hidden injuries? Researching the experiences of older men in English prisons. The Howard Journal of Crime and Justice, 44, 399–412. 10.1111/j.1468-2311.2005.00380.x

[igag075-B11] du Toit S. H. J. , Withall A., O’Loughlin K., Ninaus N., Lovarini M., Snoyman P., Butler T., Forsyth K., Surr C. A. (2019). Best care options for older prisoners with dementia: A scoping review. International Psychogeriatrics, 31, 1737–1751. 10.1017/S1041610219000681 31412973

[igag075-B12] European Prison Officers for the 21st Century. (2019-2022). https://prisonsystems.eu/projects/po21/

[igag075-B13] Eymard A. S. , Crawford B. D., Keller T. M. (2010). “Take a walk in my shoes”: Nursing students take a walk in older adults’ shoes to increase knowledge and empathy. Geriatric Nursing, 31, 137–141. 10.1016/j.gerinurse.2010.02.008

[igag075-B14] Farkas M. A. , Manning P. K. (1997). The occupational culture of corrections and police officers. Journal of Crime and Justice, 20, 51–68. 10.1080/0735648X.1997.9721581

[igag075-B15] Fineman S. (2008). The emotional organization: Passions and power. Blackwell.

[igag075-B16] Gerhardy T. H. , Schlomann A., Wahl H. W., Schmidt L. I. (2022). Effects of age simulation suits on psychological and physical outcomes: A systematic review. European Journal of Ageing, 19, 953–976. 10.1007/s10433-022-00722-1 36506694 PMC9729636

[igag075-B17] Hochschild A. R. (1983). The managed heart: Commercialization of human feeling. University of California Press.

[igag075-B18] Humblet D. (2020). Locking out emotions in locking up older prisoners? Emotional labour of Belgian prison officers and prison nurses. International Journal of Law, Crime and Justice, 61, 100376. 10.1016/j.ijlcj.2020.100376

[igag075-B19] Humblet D. (2021). The older prisoner. Palgrave MacMillan.

[igag075-B20] Humblet D. (2025a). Eindrapport: Evaluatie van de ervaringsgerichte opleiding ‘Werken met Ouderen in Detentie’. Vrije Universiteit Brussel. https://researchportal.vub.be/en/publications/eindrapport-evaluatie-van-de-ervaringsgerichte-opleiding-werken-m/

[igag075-B21] Humblet D. (2025b). *Ageism in prison*. In Snacken S., Cliquennois G., Durnescu I., Humblet D., Larrauri E. (Eds.), The Routledge handbook of European penology. Routledge.

[igag075-B22] Humblet D. , Decorte T. (2013). Detentiebeleving door oudere gevangenen in België: Een exploratief onderzoek. Panopticon, 34, 267–283. https://biblio.ugent.be/publication/3238404

[igag075-B23] Humblet D. , Naessens L., Lahaye H., Peersman W., Gillis K. (2021). Development of a strategy to raise awareness of the physical, social, and mental needs of older adults in prison. Advancing Corrections Journal, 12, 135–150. 10.65264/LPLJ3174

[igag075-B24] Inglis M. , Tully T. (2016). Elderly prison inmates: Specifying priorities for care and staff training. Evidence Based Practice Quarterly, 1, 1–9.

[igag075-B25] Iwasaki M. , Jones J. A. (2008). Attitudes toward older adults: A reexamination of two major scales. Gerontological Geriatrics Education, 29:139–157. 10.1080/02701960802223209 19042232

[igag075-B26] Jameton A. (1984). Nursing practice: The ethical issues. Prentice-Hall.

[igag075-B27] Jeong H. , Kwon H. (2021). Long-term effects of an aging suit experience on nursing college students. Nurse Education in Practice, 50, 102923. 10.1016/j.nepr.2020.102923 33333338

[igag075-B28] Jeong H. S. , Lee Y., Kwon H. (2017). Effects of senior simulation program for nursing students: An integrated study in South Korea. Eurasia Journal of Mathematics, Science and Technology Education, 13, 4437–4447. 10.12973/eurasia.2017.00936a

[igag075-B29] Kogan N. (1961). Attitudes toward old people: The development of a scale and an examination of correlates. Journal of Abnormal and Social Psychology, 62, 44–54. 10.1037/h0042000 13757539

[igag075-B30] Kolb D. A. (1984). Experiential learning: Experience as the source of learning and development. Prentice-Hall.

[igag075-B31] Loeb S. J. , Murphy J. L., Kitt-Lewis E., Wion R. K., Jerrod T., Myers V. H. (2021). Inmates care: Computer-based training for geriatric and end-of-life care in prisons. Journal of Correctional Health Care, 27, 132–144. 10.1089/jchc.20.03.0016 34232784 PMC8459735

[igag075-B32] Masters J. L. , Magnuson T. M., Bayer B. L., Potter J. F., Falkowski P. P. (2016). Preparing corrections staff for the future. Journal of Correctional Health Care, 22, 118–128. 10.1177/1078345816634667 26984135

[igag075-B33] McLaren H. , Richards J., Patmisari E. (2025). A concept evolution inspired by Delphi: The quest for a fair and dignified model for ageing in prison. Journal of Aging Studies, 75, 101378. 10.1016/j.jaging.2025.101378 41352893

[igag075-B34] MenACE (2018). *MenACE State of the Art Report and Existing Practices Review*. [Report]. http://www.menace-project.org

[igag075-B35] Mezirow J. (1991). Transformative dimensions of adult learning. Jossey-Bass.

[igag075-B36] Mezirow J. (1997). Transformative learning: Theory to practice. New Directions for Adult and Continuing Education, 74, 5–12. 10.1002/ace.7401

[igag075-B37] Miller K. E. M. , Shen K., Yang Y., Williams B. A., Wolff J. L. (2024*).* Prevalence of disability among older adults in prison. JAMA Network Open, 7(12), e2452334. 10.1001/jamanetworkopen.2024.52334 39729319 PMC11681372

[igag075-B38] Momtaz Y. A. , Fadayevatan R., Mollaei P., Mousavi-Shirazi F. (2018). Development and validation of the Willingness to Work with Elderly People Scale (WEPS) among medical sciences students. Clinical Gerontologist & Geriatrics, 9, 149–155. 10.33879/JCGG.2018.1815

[igag075-B39] Novisky M. A. , Prost S. G., Fleury-Steiner B., Testa A. (2025). Linkages between incarceration and health for older adults. Health Justice, 13, 23. 10.1186/s40352-025-00331-x 40244545 PMC12004771

[igag075-B40] Perot J. G. , Jarzebowski W., Lafuente-Lafuente C., Crozet C., Belmin J. (2020). Aging-simulation experience: Impact on health professionals’ social representations. BMC Geriatrics, 20, 14. 10.1186/s12877-019-1409-3 31964337 PMC6975088

[igag075-B41] Riess H. (2017). The science of empathy. Journal of Patient Experience, 4, 74–77. 10.1177/2374373517699267 28725865 PMC5513638

[igag075-B42] Sundt J. , Reiter K., Williams B. A. (2024). The interdependence of caring, safety, and health in correctional settings: Analysis of a survey of security staff in a large county jail system. Social Science & Medicine, 358, 117218. 10.1016/j.socscimed.2024.117218 39178533

[igag075-B43] Tremayne P. , Burdett J., Utecht C. (2011). Simulation suit aids tailored care. Nursing Older People, 23, 19–22. 10.7748/nop2011.09.23.19.c8678 21980792

[igag075-B44] Villaumé A. , Artico R., Souques C., Cedat C., Mébasti A., Gouveia N., Duron E., Karoubi E. (2019). Perception du vieillissement chez les soignants en gériatrie après mise en situation avec une combinaison simulant le grand âge/ Perception of aging among geriatric health professionals after role play staging old age. NPG – Neurologie, Psychiatrie, Gériatrie, 19, 333–339. 10.1016/j.npg.2019.09.002

[igag075-B45] Wilkinson D. , Caulfield L. (2020). A systematic review of the characteristics and needs of older prisoners. Journal of Criminal Psychology, 10, 253–276. 10.1108/JCP-06-2020-0023

[igag075-B46] Williams B. A. , Lindquist K., Hill T., Baillargeon J., Mellow J., Greifinger R., Walter L. C. (2009). Caregiving behind bars: Correctional officer reports of disability in geriatric prisoners. Journal of American Geriatrics Society, 57:1286–1292. 10.1111/j.1532-5415.2009.02286.x 19582902

