# Supplementary material for: Walk a mile in my old shoes: an intervention study on age simulation suit training for correctional staff
Source: Innov Aging. 2026 Jun 29;10(9):igag075. doi: 10.1093/geroni/igag075 (PMC13453337; doi:10.1093/geroni/igag075)
Supplement: Supplementary file 1 [file igag075_supplementary_data.pdf]

***Innovation in Aging* Supplementary Material: Humblet. Walk a mile in my old shoes: An intervention study on age simulation suit training for correctional staff.**

Supplementary Text 1: Methods

Details on the Geriatric Care Training Module

The module is comprised of a classical in-person training, which aims to (1) understand the characteristics of older adults in prison, (2) comprehend the ageing process, (3) identify the specific needs of people ageing in prison, (4) gain insight into their life histories, and (5) recognize the significance of small but meaningful actions. The sixth objective, i.e. experiencing the perspective of an older person in prison, is addressed through the simulation scenarios.

Role-playing Scenarios (translated)

Scenarios Based on Literature and Consultations with Trainers from the Training Centre for Penitentiary Staff

Scenario 1 –

A person in detention is seated in a wheelchair in their cell. They attempt to drink a glass of water or have coffee and try to prepare a sandwich, requesting assistance from the prison officer. The prison officer responds that there is sufficient time and that the person should continue attempting independently. Meanwhile, the prison officer engages in conversation with a colleague or walks away.

Scenario 2 –

The person in detention is asked to clean the cell. Subsequently, the prison officer inspects the work. The person has fallen and may be lying on the floor and is expected to rise independently or may be unable to reach the alarm button, leaving them to wait until the prison officer passes by. The person later wishes to make a phone call to request a meeting with their lawyer/family member the following day.

Scenario 3 –

The person in detention is asked to prepare for outdoor time or a visit. They are required to put on shoes, tie laces, and dress appropriately. To reach the outdoor area or visiting room, they must navigate stairs. They undergo a standard body search, during which staff exchange a cynical remark: "Got time? He'll get there eventually…"

Scenario 4 –

The person in detention is asked to walk the stairs (one time without help from the prison officer and one time with help from the prison officer) and to move into a shared cell and climb to the upper bunk assigned by their cellmate.

Prison officer = Participant assuming the role of prison officer
Person in detention = Participant assuming the role of an older detainee, wearing parts of the GERT suit (age-simulation suit)

GERT Age Simulation Suit

The GERT suit was selected because it offers a scientifically validated, modular design that simulates a wide range of sensory and motor impairments associated with ageing. Compared to other ageing suits, it has been widely applied in professional training contexts (e.g., healthcare, education) and demonstrated effectiveness in fostering empathy and awareness of age-related limitations (see: www.age-simulation-suit.com).

Instrument Adaptation Process

The instruments were adapted based on sessions with trainers to ensure relevance to the specific context (prison setting) and were subsequently translated into Dutch and back-translated. This process ensured that the items were culturally and contextually appropriate for the study population, while maintaining as much as possible the original constructs of the scales. However, a psychometric validation was not conducted. As a result, the adapted Dutch versions of the instruments should be interpreted with caution, as their reliability and validity in this specific context have not been established. The pre- and post-questionnaires can be openly accessed via the institutional repository (https://researchportal.vub.be/en/publications/eindrapport-evaluatie-van-de-ervaringsgerichte-opleiding-werken-m/).

Supplementary Table 1: Selected items of the Kogan scale

1. “It is probably much better for older people to live in separate units with people of their own age.”
2. “Most older people have ways of being and/or behaving that are difficult to change.”
3. “Most older people can adapt when the situation requires it.” (reverse-coded)
4. “It is foolish to think that wisdom comes with age.” (reverse-coded)
5. “People become wiser with age.”
6. “Most older people make others feel uncomfortable.” (reverse-coded)
7. “Most older people make others feel at ease when they are with them.”
8. “There are exceptions, but generally most older people are quite alike.” (reverse-coded)
9. “It is clear that most older people are very different from one another.”
10. “Most older people should be more concerned about their personal appearance; they tend to be quite untidy.” (reverse-coded)

Supplementary Table 2: Background characteristics of participants

**Characteristic**	***n* (%) or Range**
Total participants	19
Gender	Male: 16 (84%), Female: 3 (16%)
Age (years)	25–64
Prison location	Merksplas: 9, Brugge: 7, Dendermonde: 2, Oudenaarde: 1
Unit type	Specialized older adult units: 16
Prior experience with older inmates	13
Previous work experience with older adults	12
Personal experience in elder care	9
Roles	Correctional officers: 17, Team leader: 1, Detention supervisor: 1
Prison experience (years)	1–5: 3, 6–10: 3, 11–20: 13, >20: 2
Education	Secondary: 11, Vocational: 4, Associate degree: 1, Bachelor: 3
Prior training on older adults in detention	9

Supplementary Table 3: Summary of role assignment and enactment during the scenarios

The table below outlines the assignment of roles and their enactment during the scenarios. Participants selected their roles voluntarily, in accordance with ethical considerations governing participation.

Session	Participants	Role(s)	Suit Worn	Observers	Notes
1	9	2 Officers, 2 Inmates, All Observers	2 wore suits	All participants observed	Only 2 participants wore suits during scenario; everyone else observed.
2	10	1 Officer, 4 Inmates, All Observers,	7 wore suits	All participants observed	Most participants tried parts of the suit (except 3), but not always during scenario; felt more comfortable wearing it in between scenarios.
Summary	19	3 Officers, 6 Inmates, 19 Observers	9 wore suits	19 Observers	Combines both sessions; suit usage higher in session 2.

**Note*: One participant (male, session 2) also wore a suit and took part in focus group discussions and sessions, but did not complete the post-measurement. This participant is included in the above counts.

Supplementary Table 4: Pre- and post-test scores with paired-samples t-test results

**Measure**	**Pre-test *M* (SD)**	**Post-test *M* (SD)**	**t(df)**	***p* (one-sided)**
Knowledge	6.42 (1.43)	7.16 (1.26)	-1.68 (18)	.055
Attitudes toward older adults	3.90 (0.24)	3.77 (0.24)	2.42 (18)	.013
Ageism (benevolent)	3.67 (0.63)	3.62 (0.72)	0.36 (17)	.361
Ageism (hostile)	3.11 (1.06)	3.18 (0.62)	-0.36 (17)	.361
WWEP – attitudes	5.23 (0.92)	5.36 (0.73)	-1.33 (17)	.101
WWEP – competence	3.92 (0.85)	4.04 (1.00)	-0.70 (17)	.247
WWEP – motivation	4.42 (1.32)	4.52 (1.19)	-0.41 (17)	.342

Supplementary Text 2: Focus group discussion guide (original language)

1. **Beschrijving van gebeurtenissen** *(voor zover dat nog niet gebeurd is voordien)*

   Laten we beginnen met de simulatie zelf. Kunnen jullie kort beschrijven wat er gebeurde tijdens de simulatie?

   - Wat viel jullie het meeste op tijdens de simulatieoefeningen – tijdens het dragen van het verouderingspak en uitvoeren van bepaalde taken?
   - Hoe beïnvloedde het verouderingspak het vermogen om te bewegen en te reageren?

2. **Emotionele betrokkenheid**

   Wat hebben jullie ervaren tijdens de simulatie?

   - Hoe voelde u zich tijdens de simulatie, vooral met betrekking tot de beperkingen die werden ervaren?

3. **Uitdagingen en lessen**

   Welke aspecten van de simulatie vond u het meest uitdagend of leerzaam?

   - Welke positieve of negatieve veranderingen hebt u ervaren na deelname aan deze training?
   - Heeft deze ervaring u nieuwe inzichten gegeven die u niet verwachtte?

4. **Significante veranderingen**

   Welke veranderingen hebben jullie ervaren door deze simulatie? Wat is de meest belangrijke?

   - Was er een specifiek moment dat uw denken of gedrag heeft veranderd?
   - In hoeverre heeft deze ervaring jullie begrip van de uitdagingen van oudere gedetineerden veranderd?
   - Hoe heeft deze simulatie uw kijk op (de zorg en begeleiding) van oudere gedetineerden veranderd?

5. **Toepassing van de ervaring in het werk**

   Ziet u overeenkomsten met uw dagelijkse interacties met oudere personen in detentie?

   Hoe zou u deze ervaring kunnen gebruiken in uw dagelijkse werk?

   - Wat zou u in uw werkomgeving anders doen naar aanleiding van deze training?
   - Hoe denkt u dat deze simulatie uw aanpak in de omgang met ouderen in detentie zal beïnvloeden?

6. **Reflectie en toepassing**

   Hoe kunt u deze ervaring gebruiken in uw dagelijkse werk?

   - Ziet u mogelijkheden om dit in de praktijk toe te passen?
   - Wat is volgens u het belangrijkste dat u heeft geleerd, en welke invloed heeft dit op uw werk?
   - Hoe kunnen we deze training verbeteren?
